# Experimental measurement of preferences in health and healthcare using best-worst scaling: an overview

**DOI:** 10.1186/s13561-015-0079-x

**Published:** 2016-01-08

**Authors:** Axel C. Mühlbacher, Anika Kaczynski, Peter Zweifel, F. Reed Johnson

**Affiliations:** 1 IGM Institute for Health Economics and Health Care Management, Hochschule Neubrandenburg, Neubrandenburg, Germany; 2 Department of Economics, University of Zürich, Zürich, Switzerland; 3 Center for Clinical and Genetic Economics, Duke Clinical Research Institute, Duke University, Durham, USA

**Keywords:** Best-worst scaling, BWS, Experimental measurement, Healthcare decision making, Patient preferences

## Abstract

Best-worst scaling (BWS), also known as maximum-difference scaling, is a multiattribute approach to measuring preferences. BWS aims at the analysis of preferences regarding a set of attributes, their levels or alternatives. It is a stated-preference method based on the assumption that respondents are capable of making judgments regarding the best and the worst (or the most and least important, respectively) out of three or more elements of a choice-set. As is true of discrete choice experiments (DCE) generally, BWS avoids the known weaknesses of rating and ranking scales while holding the promise of generating additional information by making respondents choose twice, namely the best as well as the worst criteria. A systematic literature review found 53 BWS applications in health and healthcare. This article expounds possibilities of application, the underlying theoretical concepts and the implementation of BWS in its three variants: ‘object case’, ‘profile case’, ‘multiprofile case’. This paper contains a survey of BWS methods and revolves around study design, experimental design, and data analysis. Moreover the article discusses the strengths and weaknesses of the three types of BWS distinguished and offered an outlook. A companion paper focuses on special issues of theory and statistical inference confronting BWS in preference measurement.

## Background: preferences in healthcare decision making

The primary responsibility of healthcare decision makers is to determine the optimal allocation of scarce money, time, and technological resources, given available information on outcomes. Both regulatory and clinical healthcare decisions indirectly or directly affect the welfare of healthcare recipients. However, decision makers often lack information about how the criteria they use should be weighted from the point of view of taxpayers, insurers, and patients. For example, little is known about patients’ willingness to accept trade-offs among life-years gained, restrictions on activities of daily living, and the risk of side effects. To the extent that healthcare decision makers lack information on the preferences of those affected, resource-allocation decisions will fail to achieve optimal outcomes.

When searching for optimal solutions, decision makers therefore inevitably must evaluate trade-offs, which call for multiattribute valuation methods. In this task, discrete choice experiment (DCE) methods have proven to be particularly useful [1–5]. More recently, some researchers have proposed using best-worst scaling (BWS) methods. BWS is a variant of DCEs that seeks to obtain extra information by asking survey respondents to simultaneously identify the best and worst items in each set of scenarios (attributes, levels or alternatives).

This paper is structured as follows. In Literature review the underlying systematic review of published BWS studies in health and healthcare is described. BWS - survey of methods contains a survey of BWS methods, while Conducting a BWS experiment revolves around study design, experimental design, and data analysis. Overview of recent BWS applications discusses the strengths and weaknesses of the three types of BWS distinguished. An overview of applications of BWS is presented in Discussion: strengths and weaknesses in application. Conclusions and an outlook are offered in Conclusions and outlook. A companion paper (Mühlbacher et al. [6]) focuses on special issues of theory and statistical inference confronting BWS in preference measurement.

## Literature review

A systematic review was conducted, limited to English and German language publications in the databases ‘pubmed’ and ‘springerlink’. Overall 53 BWS applications were published in the last 10 years until September 2015. The following search terms were used for the review: ‘Best-Worst Scaling’, ‘Best-Worst Scaling AND Health*’, ‘Best Worst Scaling’, ‘Best Worst Scaling AND Health*’, ‘MaxDiff Scaling’, ‘Maximum Difference Scaling’. Data on authors, title, date, type of elicitation format, study objective, and sample size were extracted.

## BWS - survey of methods

### Microeconomic foundations

BWS as a variant of DCE starts from the basic assumption of Thurstone that individuals maximize utility, with some determinants of utility unobservable for the experimenters [7]. Hence, utility can be decomposed into a deterministic systematic and an unobservable stochastic component [8]. Furthermore, Thurstone’s law of comparative judgment calls for pairwise comparisons. Marschak and Luce extended, formalized, and axiomatized this law [9, 10]. In addition to the probit model (attributed to Thurstone), McFadden used random utility theory to derive the multinominal logit (MNL) model for estimating choice probabilities; he received the Nobel Prize in Economics for this contribution [11, 12].

### Preference measurement

Choice-based preference measurement as described above competes with two other approaches: rating (which makes survey respondents assign numerical values to alternatives), and ranking (which makes them construct a preference ordering of alternatives). Numerous studies identify preferences from respondents’ ratings, rankings, or choices. While rating techniques are critically discussed, all three approaches require basic assumptions of logic and consistency. They differ in terms of additional assumptions about preference measurability, levels of cognitive effort, and vulnerability to biases. In particular, a rating assumes utility to be a cardinally measured quantity (which it is not). As shown in BWS - survey of methods of the companion paper, ratings therefore cannot predict choice [6].

### Best-worst scaling

BWS was developed in the late 1980s as an alternative to existing approaches. Flynn distinguishes three cases of BWS which have in common that respondents, rather than just identifying the best alternative, simultaneously select the best and worst alternative from a set of three or more attributes, attribute levels or alternatives [13–15]. One of the three variants is very similar to DCEs, making it well anchored in economic theory.

### Variants of best-worst scaling

#### Object case BWS

**Fig. 1:**
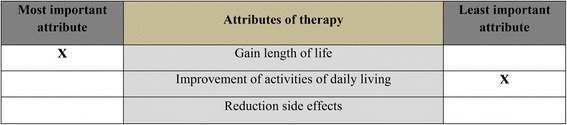
Example of an object case BWS choice scenario

The first variant of BWS is the attribute or object case. It is the original form of BWS as proposed by Finn and Louviere [16]. The object case is designed to determine the relative importance of attributes [14]. Accordingly, attributes have no (or only one) level, and choice scenarios differ merely in the particular subset of attributes shown. Figure 1 illustrates the case of three relevant attributes. Respondents are asked to identify the best and worst or the most and least preferred attribute from the set of scenarios [13]. The number of scenarios required to identify a complete ranking depends on the number of attributes. The BWS object case originally was conceived as a replacement for traditional methods of measurement such as ratings and Likert scales [14].

#### Profile case BWS

**Fig. 2:**
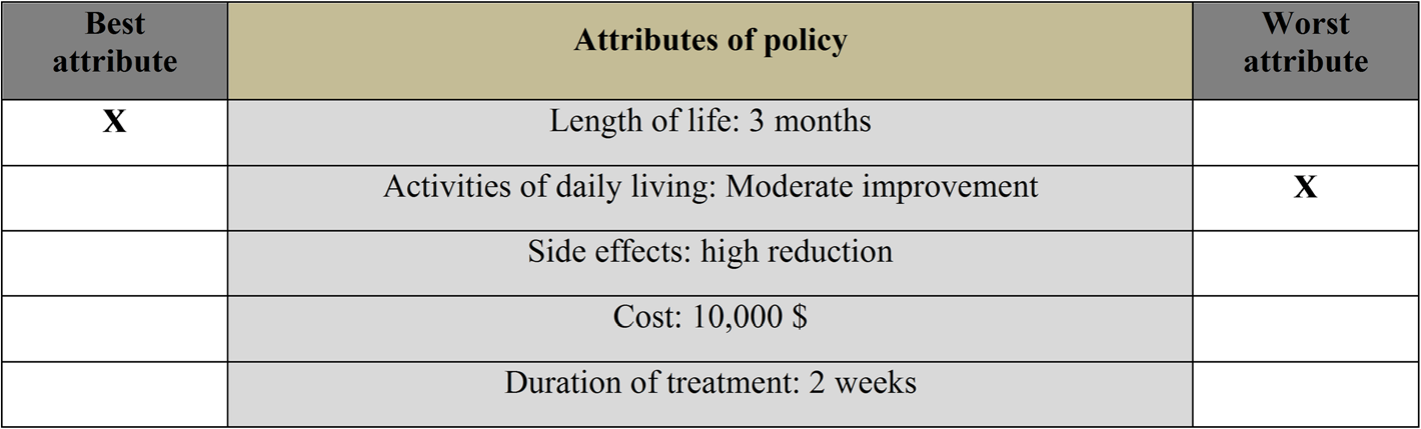
Example of a profile case BWS choice scenario

The second BWS variant is the profile case [17]. In contrast to the object case, the level of each attribute is shown. Accordingly, the same attributes appear in each scenario, while their levels change. Respondents identify both the best (most preferred) and worst (least preferred) attribute level in each scenario presented [15]. In Fig. 2 a possible healthcare intervention is characterized by five attributes: length of life, activities of daily living, side effects, cost, and duration of treatment. Profile case BWS has advantages relative to both the object case and DCEs. Contrary to object case BWS, respondents explicitly value attribute levels, making choices much more transparent and informative. Because they have to evaluate only one profile scenario at a time, constructing experimental designs is easier compared to DCEs. DCEs have to display choice sets, containing two or more choice alternatives. Therefore profiles have to be combined correctly with one or more additional profiles. Some authors argue that profile case BWS also reduces the cognitive burden of the elicitation task [17]. Accordingly, they claim that these two advantages allow an increase in the number of attributes to be valued.

#### Multiprofile case BWS

The third BWS variant is the multiprofile case [14, 18]. Contrary to the two previous cases, respondents repeatedly choose between alternatives that include all the attributes, with their levels varying in a sequence of choice sets. Thus, the multiprofile case BWS amounts to a best-worst discrete choice experiment (BWDCE). A BWDCE extracts more information from a choice scenario than a conventional DCE because it asks not only for the best (most preferred) but also the worst (least preferred) alternative. A complete ranking of more than three alternatives requires the exclusion of alternatives already identified as best and worst and asking the same question again with reference to the reduced choice set [13].

**Fig. 3:**
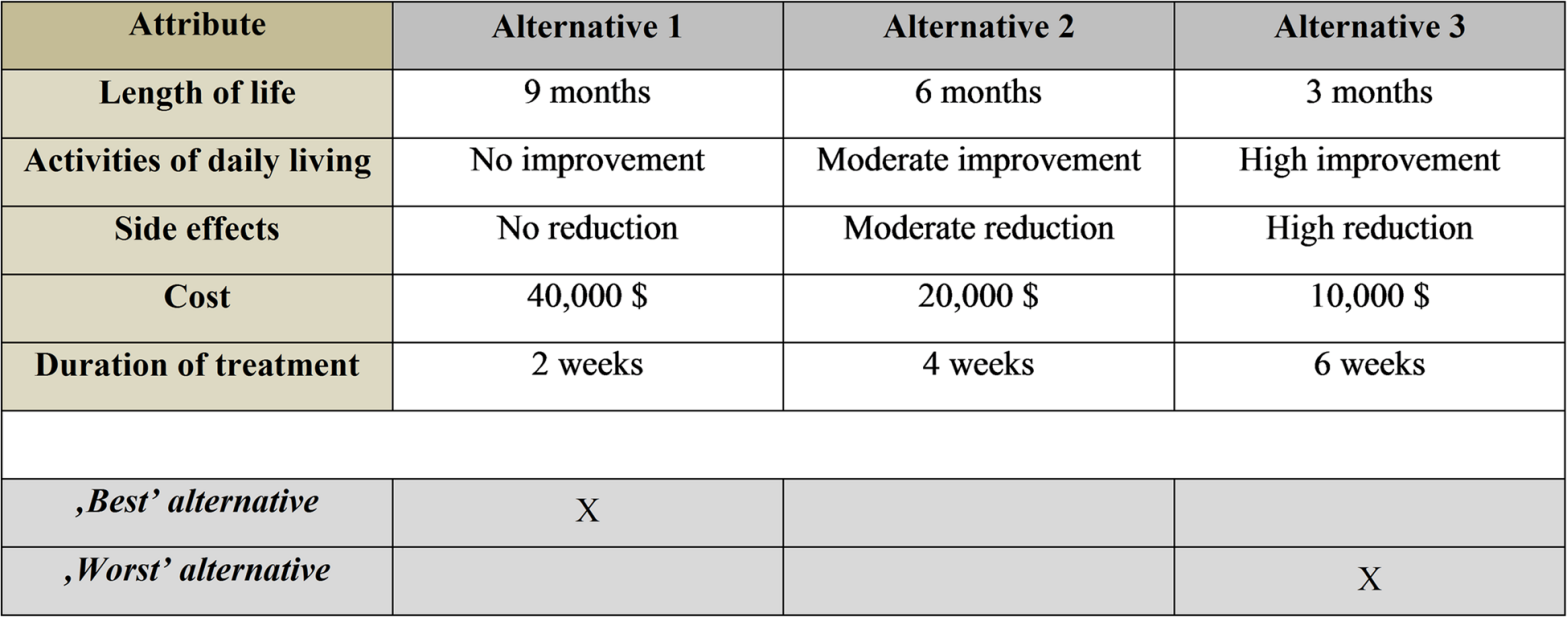
Example of a multiprofile case BWS choice scenario

An example choice scenario is shown in Fig. 3, taking again a healthcare intervention as the example. Respondents now need to evaluate five attribute levels to identify alternatives as best and worst, respectively. The fact that the respondent shown considers alternative 1 as the worst indicates that he or she does not value length of life quite so highly but dislikes the personal cost of treatment. Conversely, by identifying alternative 3 as best, the same respondent indicates that he or she would be interested in improving activities of daily living or reducing cost, while length of life is relatively less important (otherwise, policy 3 would have been more preferred).

## Conducting a BWS experiment

### Study design

The first step in conducting a BWS experiment is to state the research question and to define the decision problem, with the objective of identifying the set of relevant attributes. This calls for a comprehensive literature search in addition to expert surveys, personal interviews, and pre-tests (which usually involve interviews or focus groups) [19]. Several requirements need to be met. First, the attributes and attribute levels selected should be relevant to respondents while still being under control by the relevant decision makers. Second, they should be in a substitutive relationship (otherwise no trade-offs are required), not lexicographic (otherwise no trade-offs are accepted), lack dominance (for the same reason), and be clearly defined [20]. Finally, they need to be sufficiently realistic to ensure that respondents take the experiment seriously.

#### Attributes and levels

Several methods are available for choosing attributes that can be used in combination. Direct approaches include the elicitation technique, the repertory grid method, as well as directly asking for attributes relative subjective importance. All essential attributes should appear in the choice scenarios to avoid specification error in estimating the utility function.

With the relevant attributes identified, their levels need to be defined (at least for BWS profile and multiprofile cases). Their ranges should represent the perceived differences in respondents’ utility associated with the most and least preferred level. However, the reverse is not true: A respondent’s maximum difference in utility may fall short of or exceed the spread between levels as imposed by the experiment. Also, requiring attribute levels to be realistic appears intuitive. Yet, the experiment may call for a spreading of levels, especially in an attribute whose (marginal) utility is to be estimated with high precision (this is the price attribute if willingness-to-pay values are to be calculated).

#### Alternatives

Defining full attribute-level descriptions is required only for multiprofile case BWS, where respondents are asked to evaluate alternatives. If one were to present them with all possible combinations, they would have to deal with hundreds, even thousands of combinations. For instance, four attributes with five levels each already result in as many as 5^4^ = 625 combinations – too many to handle for any respondent. However, this number can be reduced using a method analogous to principal-component analysis, at the price of a certain loss of information (for more details, see [2, 4]). In healthcare, the alternatives could represent different health technologies, treatments, or ways of providing care, characterized by varying attribute levels (see Fig. 2).

### Experimental design

Survey design involves constructing scenarios comprising combinations of attributes or attribute levels. As in the case of a DCE, there are several options available for BWS. The full-factorial design only can be used for a maximum of three attributes with three levels each, the number of scenarios attaining already 3^3^ = 27. In all other cases, a fractional factorial design is advisable. Here, the selection of scenarios is structured to generate the maximum amount of information. Thus scenario selection depends critically on relationships among attributes [21]. Several options are described in more detail below, with no one dominating the other two in terms of all criteria [22].

#### Manual design

From a complete list of possible combinations, suitable designs can be created manually by judiciously balancing several criteria, viz. the number of scenarios involving high and low (assumed) utility values, low correlation of attributes (orthogonality), balanced representation, and minimum overlap of levels [23]. If the reduced number of choice scenarios to be presented to respondents turns out to be still excessive, design blocks have to be created. For example, one-half of the respondents are assigned to one block of scenarios while the other half is assigned to another block. Assignment of respondents to blocks should be random to avoid selection bias.

A frequently used alternative is the Balanced Incomplete Block Design (BIBD) [24]. Because a BIBD is subject to the symmetry requirement, the number of possible BIBDs is limited. For guidance concerning creation, analysis, and operationalization of manual designs, the main reference is Cochran and Cox, who created a multitude of ready-to-use BIBDs [25]. Ways to increase design efficiency are described in Chrzan and Orme and Louviere et al. [22, 26]. More recently, optimal and near-optimal designs complementing the manual approach have been developed [27].

#### Optimized design

Rather than manually developing a design, researchers can use automated (often computerized) procedures. For example, the software package SAS offers several search algorithms to determine the most efficient design of a given experiment [23]. Simple orthogonal main-effect design plans (OMEPs) are available as well (e.g. in SPSS). Easy to use, they have been popular in BWS.

### Data analysis

The data collected in the course of a BWS experiment can be analysed in several ways. The four most important are described in this section.

#### Count analysis

Orthogonal BWS designs can be analysed using count analysis, which is limited to examining choice frequencies. Count analysis may therefore be applied across all respondents as well as at the individual-respondent level [14]. A best-worst score can be constructed based on the difference *Total(Best) – Total(Worst)* [28].

Some authors propose taking the square root of the ratio *Total(Best)/Total(Worst)*, either at the level of a single attribute or at the level of complete decision scenarios [15].

The square root of the ratio between best and worst counts decreases as a function of *r*, the number of alternatives presented in a nonlinear, degressive way. A degree of standardization can be achieved by dividing best-worst scores by the product of the frequency of occurrence (attributes, levels, alternatives) and sample size. For more details, see in particular Louviere as well as Crouch and Louviere [29]. Count scores provide information about the importance and hierarchy of attributes but fail to ensure comparability of results across BWS studies. Specifically, no conclusions regarding the relative economic importance of attributes measured by marginal rates of substitution are possible. Recall that the subjective distance between best and worst may turn out differently because distances between best and worst are not scale-invariant (see Section 5.3 of the companion paper for details). As a consequence, questions such as whether there are differences in trade-offs between side effects and prolonging life between young and old people cannot be answered.

#### Multinomial logit, mixed logit and rank-ordered logistic regression models

One use of BWS is to determine the likelihood that an attribute, an attribute level, or an alternative is identified as most important or least important. This calls for dual coding, namely best = 1 if the attribute is chosen as the most important in the combination, and best = 0 otherwise, as well as worst = 1 if it is cited as least important, and worst = 0 otherwise. As a result, there are two variables to be analysed, both of which can only take on the values zero and one. Taking into account that 0 and 1 bound a probability, the logit procedure yields propensity scores reflecting the probability of an attribute being present in a given combination.

A linear regression also produces estimates of relative importance, which however may fall outside the allowable range bounded by zero and one and hence cannot be interpreted as choice probabilities. Some authors neglect this, applying weighted least squares. The weighting is necessary because the (0,1) property of the dependent variable causes the error term ε to have non-constant variance, violating a requirement of ordinary least squares [30]. While logit models are rooted in random utility theory and hence real-world choice behaviour, linear probability models do not bear a direct relationship with choice and decision making. Note that logit coefficients do not reflect differences in probability but need to be transformed for this purpose. Also, since a regression determines the conditional expected value of the dependent variable, it measures average preferences rather than those of an individual person [31]. By introducing interaction terms (see above), socio-economic characteristics can be taken into account, allowing for group-specific estimates. These are usually sufficient for decision-making in health policy but may be a shortcoming in a marketing context. Details can be found in Flynn et al., Flynn et al. as well as Wirth [30–32].

A popular MNL-based model for best-worst choice is the maxdiff model. The maxdiff procedure calls for identifying the maximum difference in utility. As shown by Flynn and Marley, the generalization of the MNL model assumes that the utility associated with the choice of the best option is the negative of the utility of associated with the choice of the worst option [33]. Evidently, the best-worst distance in the maxdiff formulation is expressed in terms of cardinal utility, a problematic property in view of microeconomic theory [17, 34]. Additional weaknesses include failure to determine the relative importance of attributes [for more detail, see the companion paper by Mühlbacher et al.].

The mixed logit (MXL) model overcomes some of the limitations of the MNL model. MXL estimation accommodates unobserved taste heterogeneity by specifying preference parameters as random variables with means and standard deviations rather than fixed parameters. MXL involves three main specification issues: (1) determination of the parameters that are to be modelled as random variables; (2) choice of so-called mixing distributions for the random coefficients; and (3) economic interpretation of estimated random coefficients [35–37]. In return, MXL can represent general substitution patterns because it is not subject to the restrictive independence of irrelevant alternatives (IIA) property of MNL estimation [38].

Alternatively, rank-ordered logistic regression models (ROLM) or „exploded logit“ models can be applied to Best-Worst Scaling. ROLM allow modeling the partial rankings obtained from the responses to the Best-Worst Scaling questions. This robust analysis is a generalization of the conditional logit model for ranked outcomes but does not violate the assumption of the independence of irrelevant alternatives (IIA) [39].

#### Latent class analysis

Latent class analysis, a form of cluster analysis, is particularly useful in the event that attempts at forming homogeneous groups using observable socio-economic characteristics fail [40]. For example, point estimates of marginal WTP may be scattered within a certain age group, suggesting hidden heterogeneity caused by differences in choice behavior not linked to age. MNL estimation thus needs to be generalized in ways as to be able to infer two or more latent groups of unknown size from observations.

Accordingly, along with the likelihood of a respondent belonging to a certain group, latent class analysis estimates group-specific utility functions without splitting the sample. Individual utilities associated with an alternative then can be calculated as the group-specific estimate weighted by the probability of the respondent belonging to this group [41]. Since this probability depends on the assumed number of latent groups and therefore has to be determined again and again in the course of the estimation, a large number of observations are necessary to obtain statistically significant results. In hierarchical Bayes estimation, to be described below, smaller samples are sufficient, but at the cost of more restrictive assumptions [28].

#### Hierarchical bayes estimation

Hierarchical Bayes (HB) estimation increasingly is being applied to the analysis of DCEs [38]. It fits a priori distributions of the parameters to be estimated to the sample data, using individual-specific data to derive a posteriori distributions. Prior knowledge, such as the negative sign of the coefficient pertaining to the price attribute, can be incorporated in the estimation. While the multinominal normal often is assumed for the priori distribution, the rationale for assuming normal distributions for random errors does not carry over to taste distributions. There is no reason to suppose that tastes are symmetrically distributed with infinite support. For small designs with no blocking, HB estimation can yield reliable individual best-worst values even when the number of responses per respondent is small. It is also efficient in the sense that for estimating the utility of an individual respondent, the choices of other respondents need not to be taken into account [42]. MNL estimation allows determining the choice probability for a given choice scenario. Applied to BWS data, it is very similar to HB estimation of a DCE, with the only difference that BWS additionally requires analysis of the worst choice. Since a closed solution for deriving the posteriori distribution is not available, simulation methods are required, which are supported by standard statistical software [32].

## Results and overview of recent BWS applications

**Fig. 4:**
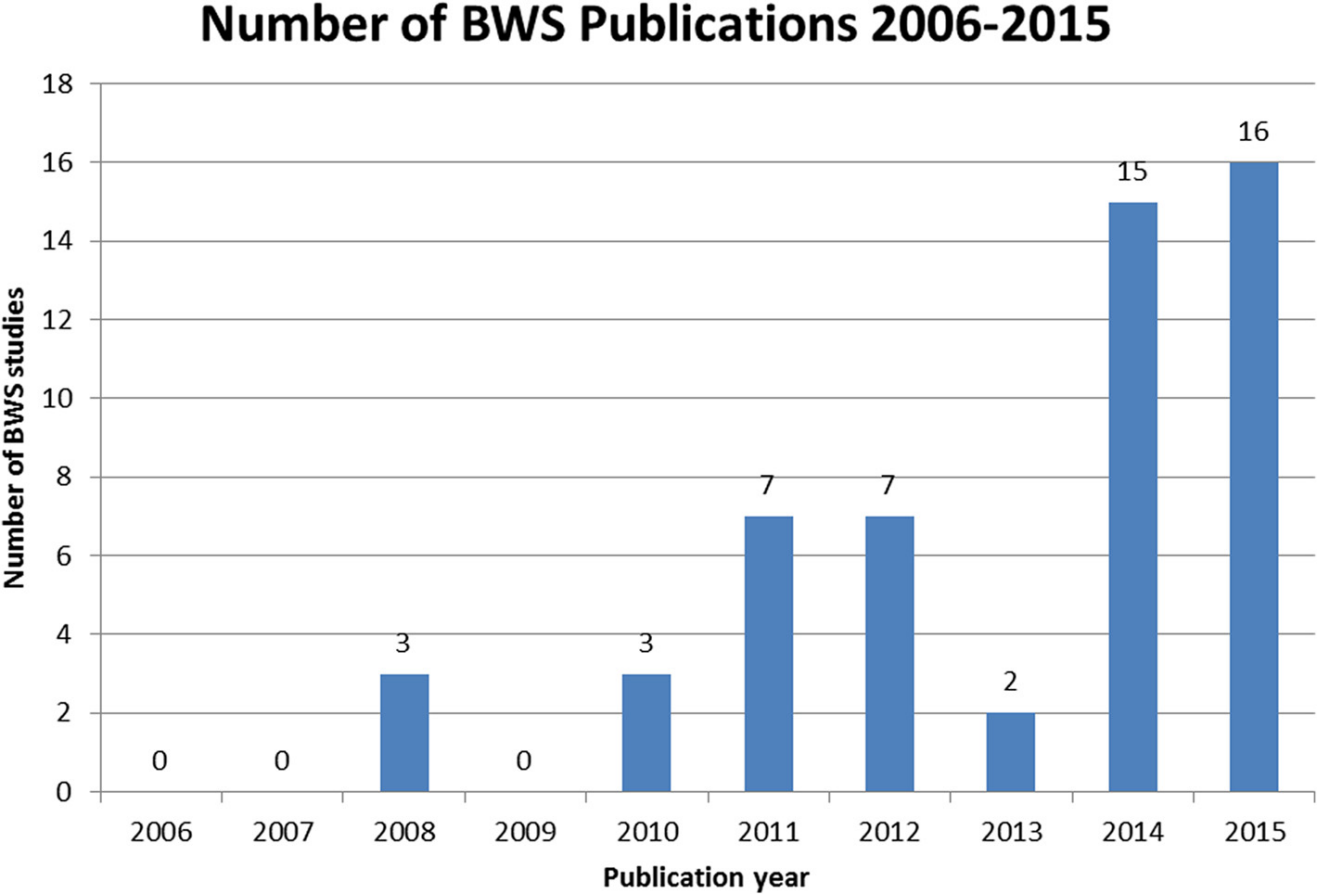
Number of BWS Publications 2006–2015

The literature search generated a total of 53 publications which met the inclusion criteria (see appendix for their listing and key characteristics). As shown in Fig. 4, there was a substantial increase in the number of BWS applications to healthcare between the years 2006 and 2015, with zero annual publications up to 2007 and around 15 recently. Therefore, their absolute number is still rather small.

A crucial aspect of constructing a BWS experiment is the variant which is used for data collection. The three BWS variants (see Variants of best-worst scaling) differ in the nature and in the complexity of the items being chosen [33]. Out of the 53 BWS publications, 24 are ‘object case’ (also called case 1) BWS, 24 are ‘profile case’ (also called case 2), and five are ‘multiprofile case’ (also called case 3 or BWSDCE) studies, respectively. Thus, studies of the ‘object case’ and ‘profile case’ have been dominant in healthcare (see also appendix).

Furthermore, sample sizes vary considerably, ranging from minimum *N* = 16 [43], to maximum *N* = 5,026 [44], with a mean of *N* = 442 respondents. As to the topics addressed by BWS papers, they fall in two main categories, value of health (derived from the valuation of health states) and value of health care or intervention (derived from the valuation of treatment characteristics or changes in attribute levels).

Only eight of the 53 publications focus on the value of health in terms of health-related quality of life: they are usually based on patient reported outcomes. By way of contrast, 31 publications use BWS for the evaluation of an intervention, usually based on clinician reported outcomes. The remaining 14 studies address policy issues, examining societal preferences. Over time, there has been an increase in the number of BWS applications revolving around patient and expert preferences (see also Fig. 4).

## Discussion: strengths and weaknesses in application

BWS has a wide range of potential applications, ranging from estimating utility functions and marginal willingness to pay associated with specific attributes and entire alternatives to predicting likely acceptance of innovative healthcare products and services. While BWS is well-established in management science and marketing, it is much less used in health economics and health services research, although there is an increasing trend.

### Latent utility scale

Flynn et al. complemented their study of patient preferences with a comparison of estimation methods, finding the results of weighted least squares to be quite comparable to those of far more demanding MNL estimation [31]. Furthermore, they claim that a traditional DCE cannot be used to assign relative utility weights to attribute levels because parameter estimates and an unobserved scale factor are confounded. If true, this would amount to a major deficiency because valuation of attribute levels such as the difference in length of life between 3 months and 9 months plays an important role in utility assessment and health policy. Moreover, a marginal change in levels often needs to be evaluated against a discrete change such as the presence or absence of an attribute. However, as argued in Section 5.2 of the companion paper, the alleged unobserved scale factor may be the consequence of a failure to identify preferences correctly.

### Accuracy of predictions

BWS, in particular Case 3, was found to merely constitute a refinement of a DCE, allowing for more accurate but not fundamentally different measurement of utility differences. Comparing six approaches for determining the importance of attributes Chrzan and Golovashkina conclude that BWS improves discrimination of attributes and prediction of actual decisions [45]. This finding reflects the main strength of BWS, which is the information gain achieved by collecting additional information from each question. In this way, preference structures can be determined more precisely or with equal precision but smaller sample size. Moreover, through stepwise exclusion of alternatives identified as best and worst, BWS can yield complete rather than partial rankings.

### Cognitive burden

Decreased cognitive burden placed on respondents has been cited as an advantage of BWS; however, the available evidence is not conclusive [46]. It seems reasonable to assume that it is easier for respondents to identify two extreme points than to select the most preferred alternative in complex decision scenarios comprising two or more alternatives with many attributes, or to determine a complete hierarchy of attributes [16]. However, this argument refers to profile case BWS, which is found to lack the important property of scale invariance (see Sections 2.4.2 and 5.3 of the companion paper). The same caveat applies to the claim that BWS enables identifying individual preference scales.

### Endogenous censoring

BWS can be seen as partial rankings of attributes or levels based on sequential choices, causing the first response to have an influence on that to the second question. While this endogenous censoring changes the value of expected utility (EU), it does not affect actual choice. Consider the following example. In the first round, a respondent has to choose among {Worst1, F, G, Best1}, with F dominated by G. He or she calculates EU as the weighted sum of the utilities associated with these four outcomes, with the weights given by the (exogenous) probabilities of their occurrence. In the second round, the choice set is reduced to {F, G, Best_1_}, making the respondent calculate EU over three outcomes only, with changed probability weights. However, these weights are now endogenous because they depend on the respondent’s choice in the first round. This constitutes a violation of expected utility theory. Moreover, the second-round EU value will generally differ from the first-round one. Yet, given that Best_1_ evidently dominates G, the final choice of ‘best’ will not be affected.

### Lexicographic preferences

A BWS task simply involves identifying the most or least important decision criteria and selecting the attribute, level, or alternative which is considered to be best or worst for that attribute. BWS asks the decision maker to rank the attributes (or levels) in order of importance. For example, in choosing a specific treatment option, any increase in length of life could be more important than any improvement in activities of daily living or any reduction in side effects. In this case the decision maker employs a non-compensatory heuristic that rejects trade-offs among attributes. This lexicographic strategy involves little effort to evaluate preference-elicitation tasks. Where information is limited or when one attribute actually is considerably more important than all others, non-compensatory responses can be a valid expression of preferences. However, with greater attention to the preference-elicitation task, decision makers might have accepted lower levels of the dominant attribute in return for higher levels of other attributes. Unfortunately, as in traditional DCEs, it usually is impossible to determine whether non-compensatory responses are a valid expression of preferences or a simplifying heuristic designed to avoid the effort of evaluating trade-offs.

### Judgment versus choice

The extra information obtained by BWS may not be as valuable as claimed by some authors. Asking for the best and worst attributes provides no information about the attractiveness of the choice scenario itself, precluding predictions of effective use or demand by patients and consumers. For example, respondents who consider all options of the choice scenario as important or unimportant have no way of expressing this in responses to preference-elicitation questions. One solution is to add an opt-out or no-purchase option relative to a benchmark alternative, such as, “Is this treatment better than your current treatment?” [30].

Nevertheless, being an extension of DCEs, BWS does have advantages over traditional methods of preference measurement such as rating or ranking. But these advantages derive from the fact that the DCE is firmly anchored in economic theory, ensuring that respondents evaluate trade-offs among advantages and disadvantages of alternatives. Besides advantages, BWS also has disadvantages, which ultimately relate to the fact that additional experimental information comes at a cost. Specifically, BWS increases the time respondents need for evaluating alternatives [40, 45], casting doubt on its alleged cognitive simplicity [15]. Moreover, respondents are not guided by a predetermined scale as in rating, and they are required to make a forced decision [47]. Yet forced choices are not always unrealistic, because in many health contexts, “no treatment” is not an acceptable option. They can always be avoided if necessary by including an opt-out or no-purchase alternative in the study design.

## Conclusions and outlook

BWS has been shown to provide results of comparable reliability as DCEs, regardless of design and sample size [13, 18]. Thus BWS, particularly multiprofile case BWS, is best viewed as a refinement of the conventional DCE which opens up several new opportunities in health economics and health services research. In particular, increased preference information from each respondent facilitates identification of preference heterogeneity among respondents through including interaction terms in the regression equation (see Discussion: strengths and weaknesses in application of the companion paper).

There are several open questions that should be addressed in future research. According to Flynn and colleagues [30], there is no general basis for determining sufficient sample size for a BWS study (which is true of DCEs as well), even though there are some guidelines (see e.g. Johnson et al. or Yang J.-C. et al. [48, 49]). Also, modelling the random component of the utility function with data on best and worst choices is an important research challenge. Another question is whether socio-economic characteristics can be introduced through interaction terms as in a DCE. Best responses might depend on age, gender, and income in ways different from worst responses. This is of importance because health policy makers need to know whether the priorities of citizens vary with their socio-economic characteristics [14]. As an additional complication, attribute values could depend on the levels of other attributes, as predicted by the convexity of the indifference curves. Such dependencies have been little explored to date, not least because the samples were too small for accurate estimation of the corresponding coefficients. The additional information generated by BWS could facilitate more complex model specifications.

Physicians, researchers, and regulators often are poorly informed about advantages and limitations of stated-preference methods. Despite the increased commitment to patient-centeredness, healthcare decision makers do not fully realize that knowledge of the subjective relative importance of outcomes to those affected is needed to maximize the health benefits of available healthcare technology and resources. Therefore, establishing stated-preference data as an essential, valid component of the evidence base used to assess therapeutic options should be of high priority in health economic and health services research.

## Appendix

**Table 1:** BWS applications in healthcare 2006-2015

Author	Title	Year	BWS variant	Application area	Sampleize
Beusterien K, Kennelly MJ, Bridges JF, Amos K, Williams MJ, Vasavada S [50]	Use of best-worst scaling to assess patient perceptions of treatments for refractory overactive bladder.	2015	Object case	Evaluation of treatment options	*N* = 245
Flynn TN, Huynh E, Peters TJ, Al-Janabi H, Clemens S, Moody A, Coast J [51]	Scoring the ICECAP- A capability instrument. Estimation of a UK general population tariff	2015	Profile case	Evaluation of health state measurements	*N* = 413
Franco MR, Howard K, Sherrington C, Ferreira PH, Rose J, Gomes JL, Ferreira ML [52]	Eliciting older people’s preferences for exercise programs: a best-worst scaling choice experiment.	2015	Profile case	Evaluation of non-pharmaceutical treatments	*N* = 220
Gallego G, Dew A, Lincoln M, Bundy A, Chedid RJ, Bulkeley K, Brentnall J, Veitch C [53]	Should I stay or should I go? Exploring the job preferences of allied health professionals working with people with disability in rural Australia.	2015	Multiprofile case	Evaluation of workforce preferences in healthcare	*N* = 199 Response rate 51 %
Hashim H, Beusterien K, Bridges JFP, Amos K, Cardozo L [54]	Patient preferences for treating refractory overactive bladder in the UK	2015	Object case	Evaluation of treatment options	*N* = 139
Hollin IL, Peay HL, Bridges JF [55]	Caregiver preferences for emerging duchenne muscular dystrophy treatments: a comparison of best-worst scaling and conjoint analysis.	2015	Profile case	Evaluation of treatment options	N/A
Meyfroidt S, Hulscher M, De Cock D, Van der Elst K, Joly J, Westhovens R, Verschueren P [56]	A maximum difference scaling survey of barriers to intensive combination treatment strategies with glucocorticoids in early rheumatoid arthritis.	2015	Object case	Evaluation of treatment options	*N* = 66
Morrison W, Womer J, Nathanson P, Kersun L, Hester DM, Walsh C, Feudtner C [57]	Pediatricians’ Experience with Clinical Ethics Consultation: A National Survey	2015	Multiprofile case	Evaluation of working experiences	*N* = 659
Mühlbacher AC, Bethge, Kaczynski A, Juhnke C [58]	Objective Criteria in the Medicinal Therapy for Type II Diabetes: An Analysis of the Patients’ Perspective with Analytic Hierarchy Process and Best-Worst Scaling	2015	Profile case	Evaluation of treatment preferences	*N* = 388
Narurkar V, Shamban A, Sissins P, Stonehouse A, Gallagher C [59]	Facial treatment preferences in aesthetically aware women	2015	Object case	Evaluation of aesthetic surgeries	*N* = 603
O’Hara NN, Roy L, O’Hara LM, Spiegel JM, Lynd LD, FitzGerald JM, Yassi A, Nophale LE, Marra CA [60]	Healthcare worker preferences for active tuberculosis case finding programs in South Africa: a best-worst scaling choice experiment.	2015	Profile case	Evaluation of screening interventions	*N* = 125 Response rate 82 %
Peay HL, Hollin IL, Bridges JFP [61]	Prioritizing Parental Worry Associated with Duchenne Muscular Dystrophy Using Best-Worst Scaling	2015	Object case	Evaluation of disease effects	*N* = 119
Ratcliffe J, Huynh E, Stevens K, Brazier J, Sawyer M, Flynn T [62]	Nothing about us without us? A comparison of adolescent and adult health-state values for the child health utility-9D using profile case Best-Worst Scaling	2015	Profile case	Evaluation of health state values	N/A
Ross M, Bridges JF, Ng X, Wagner LD, Frosch E, Reeves G, dosReis S [63]	A best-worst scaling experiment to prioritize caregiver concerns about ADHD medication for children.	2015	Object case	Evaluation of a treatment option	*N* = 46
Wittenberg E, Bharel M, Saada A, Santiago E, Bridges JF, Weinreb L [64]	Measuring the Preferences of Homeless Women for Cervical Cancer Screening Interventions: Development of a Best-Worst Scaling Survey.	2015	Object case	Evaluation of Screening Interventions	N/A
Yan K, Bridges JF, Augustin S, Laine L, Garcia-Tsao G, Fraenkel L [65]	Factors impacting physicians’ decisions to prevent variceal hemorrhage.	2015	Object case	Evaluation of treatment preferences	*N* = 110
Damery S, Biswas M, Billingham L, Barton P, Al-Janabi H, Grimer R [66]	Patient preferences for clinical follow-up after primary treatment for soft tissue sarcoma: a cross-sectional survey and discrete choice experiment.	2014	Multiprofile case	Evaluation of follow-up interventions	*N* = 132 Response rate 47 %
dosReis S, Ng X, Frosch E, Reeves G, Cunningham C, Bridges JF [67]	Using Best-Worst Scaling to Measure Caregiver Preferences for Managing their Child’s ADHD: A Pilot Study.	2014	Profile case	Evaluation of a treatment option	N = 21 (development) *N* = 37 (pilot)
Ejaz A, Spolverato G, Bridges JF, Amini N, Kim Y, Pawlik TM [68]	Choosing a cancer surgeon: analyzing factors in patient decision making using a best-worst scaling methodology.	2014	Object case	Evaluation of treatment options	*N* = 214 Response rate 82 %
Hauber AB, Mohamed AF, Johnson FR, Cook M, Arrighi HM, Zhang J, Grundman M [69]	Understanding the relative importance of preserving functional abilities in Alzheimer’s disease in the United States and Germany.	2014	Object case	Evaluation of preventing treatments	*N* = 403 US *N* = 400 German
Hofstede SN, van Bodegom-Vos L, Wentink MM, Vleggeert-Lankamp CL, Vliet Vlieland TP, Marang-van de Mheen PJ; DISC study group [70]	Most important factors for the implementation of shared decision making in sciatica care: ranking among professionals and patients.	2014	Object case	Evaluation of patient-oriented methods	*N* = 246 professionals *N* = 155 patients
Peay HL, Hollin I, Fischer R, Bridges JF [71]	A community-engaged approach to quantifying caregiver preferences for the benefits and risks of emerging therapies for Duchenne muscular dystrophy.	2014	Profile case	Evaluation of treatment options	*N* = 119
Roy L MC, Bansback N, Marra C, Carr R, Chilvers M, Lynd LD [72]	Evaluating preferences for long term wheeze following RSV infection using TTO and best-worst scaling	2014	Profile case	Evaluation of disease effects	*N* = 1000 (recruited)
Torbica A, De Allegri M, Belemsaga D, Medina-Lara A, Ridde V [73]	What criteria guide national entrepreneurs’ policy decisions on user fee removal for maternal health care services? Use of a best–worst scaling choice experiment in West Africa	2014	Object case	identify criteria guiding political decisions	*N* = 89
Ungar WJ, Hadioonzadeh A, Najafzadeh M, Tsao NW, Dell S, Lynd LD [74]	Quantifying preferences for asthma control in parents and adolescents using best-worst scaling	2014	Object case	Evaluation of a treatment option	*N* = 50 parents *N* = 51 asthma-affected adolescents
van Til J, Groothuis-Oudshoorn C, Lieferink M, Dolan J, Goetghebeur M [75]	Does technique matter; a pilot study exploring weighting techniques for a multi-criteria decision support framework	2014	Object case	Evaluation of societal preferences for reimbursement decisions of a health innovation	*N* = 60
Whitty JA, Ratcliffe J, Chen G, Scuffham PA [76]	Australian public preferences for the funding of new health technologies: a comparison of discrete choice and profile case best-worst scaling methods	2014	Profile case	Evaluation of public preferences for funding decisions	*N* = 930
Whitty JA, Walker R, Golenko X, Ratcliffe J [77]	A think aloud study comparing the validity and acceptability of discrete choice and best worst scaling methods	2014	Profile case	Evaluation of preferences for healthcare in a priority-setting context	*N* = 24
Xie F, Pullenayegum E, Gaebel K, Oppe M, Krabbe PF [78]	Eliciting preferences to the EQ-5D-5 L health states: discrete choice experiment or multiprofile case of best–worst scaling?	2014	Multiprofile case	Evaluation of health state measurements	*N* = 100
Xu F, Chen G, Stevens K, Zhou H, Qi S, Wang Z4, Hong X, Chen X, Yang H, Wang C, Ratcliffe J [79]	Measuring and valuing health-related quality of life among children and adolescents in mainland China--a pilot study	2014	Profile case	Evaluation of health-related quality of life	*N* = 815
Yuan Z, Levitan B, Burton P, Poulos C, Brett Hauber A, Berlin JA [80]	Relative importance of benefits and risks associated with antithrombotic therapies for acute coronary syndrome: patient and physician perspectives.	2014	Object case	Evaluation of a treatment option	*N* = 206 patients *N* = 273 physicians
Severin F, Schmidtke J, Mühlbacher A, Rogowski WH [46]	Eliciting preferences for priority setting in genetic testing: a pilot study comparing best-worst scaling and discrete-choice experiments	2013	Profile case	Evaluation of diagnosis intervention	*N* = 26
Yoo HI, Doiron D [81]	The use of alternative preference elicitation methods in complex discrete choice experiments	2013	Profile case and multiprofile case	Evaluation of workforce preferences in healthcare	N/A
Gallego G, Bridges JF, Flynn T, Blauvelt BM, Niessen LW [82]	Using best-worst scaling in horizon scanning for hepatocellular carcinoma technologies	2012	Object case	Evaluation of diagnosis intervention	*N* = 120 Response rate 37 %
Knox SA, Viney RC, Street DJ, Haas MR, Fiebig DG, Weisberg E, Bateson D [83]	What’s good and bad about contraceptive products? A best-worst attribute experiment comparing the values of women consumers and GPs	2012	Profile case	Evaluation of contraceptive products	*N* = 162
Marti J [18]	A best–worst scaling survey of adolescents’ level of concern for health and non-health consequences of smoking	2012	Object case	Evaluation of effects of smoking	*N* = 376
Molassiotis A, Emsley R, Ashcroft D, Caress A, Ellis J, Wagland R, Bailey CD, Haines J, Williams ML, Lorigan P, Smith J, Tishelman C, Blackhall F [84]	Applying Best-Worst scaling methodology to establish delivery preferences of a symptom supportive care intervention in patients with lung cancer	2012	Profile case	Evaluation of treatment options	*N* = 87
Netten A, Burge P, Malley J, Potoglou D, Towers AM, Brazier J, Flynn T, Forder J, Wall B [85]	Outcomes of social care for adults: developing a preference-weighted measure.	2012	Profile case	Evaluation of social care outcome	*N* = 500 general population *N* = 458 people using equipment services
Ratcliffe J, Flynn T, Terlich F, Stevens K, Brazier J, Sawyer M [86]	Developing adolescent-specific health state values for economic evaluation: an application of profile case best-worst scaling to the Child Health Utility 9D	2012	Profile case and multiprofile case	Evaluation of health state values	*N* = 590
van der Wulp I, van den Hout WB, de Vries M, Stiggelbout AM, van den Akker-van Marle EM [87]	Societal preferences for standard health insurance coverage in the Netherlands: a cross-sectional study	2012	Multiprofile case	Evaluation of coverage decisions	*N* = 2000
Al-Janabi H, Flynn TN, Coast J [88]	Estimation of a preference-based carer experience scale	2011	Profile case	Evaluation of workforce preferences in healthcare	*N* = 162
Kurkjian TJ, Kenkel JM, Sykes JM, Duffy SC [89]	Impact of the current economy on facial aesthetic surgery	2011	Object case	Evaluation of economy of aesthetical surgery	*N* = 231 surgeons N/A for patients
Ratcliffe J, Couzner L, Flynn T, Sawyer M, Stevens K, Brazier J, Burgess L [43]	Valuing Child Health Utility 9D health states with a young adolescent sample: a feasibility study to compare best-worst scaling discrete-choice experiment, standard gamble and time trade-off methods	2011	Profile case	Evaluation of health state values	*N* = 16
Rudd MA [90]	An Exploratory Analysis of Societal Preferences for Research-Driven Quality of Life Improvements in Canada	2011	Object case	Evaluation of quality of life	*N* = 1920
Simon A [91]	Patient involvement and information preferences on hospital quality: results of an empirical analysis	2011	Object case	Evaluation of patient-oriented healthcare information	*N* = 276 response rate 71 %
van Hulst LT, Kievit W, van Bommel R, van Riel PL, Fraenkel L [92]	Rheumatoid arthritis patients and rheumatologists approach the decision to escalate care differently: results of a maximum difference scaling experiment	2011	Object case	Evaluation of care	*N* = 106 rheumato-logists *N* = 213 patients
Wang T, Wong B, Huang A, Khatri P, Ng C, Forgie M, Lanphear JH, O’Neill PJ [93]	Factors affecting residency rank-listing: a Maxdiff survey of graduating Canadian medical students	2011	Object case	Evaluation of workforce preferences in healthcare	*N* = 339
Günther OH, Kürstein B, Riedel-Heller SG, König HH [44]	The role of monetary and nonmonetary incentives on the choice of practice establishment: a stated preference study of young physicians in Germany	2010	Profile case	Evaluation of workforce preferences in healthcare	*N* = 5026
Imaeda A, Bender D, Fraenkel L [94]	What Is Most Important to Patients when Deciding about Colorectal Screening?	2010	Object case	Evaluation of screening interventions	*N* = 92
Louviere JJ, Flynn TN [14]	Using best-worst scaling choice experiments to measure public perceptions and preferences for healthcare reform in Australia	2010	Object case	Evaluation of healthcare system reform principles	*N* = 204 response rate 85 %
Flynn TN, Louviere JJ, Peters TJ, Coast J [31]	Estimating preferences for a dermatology consultation using best-worst scaling: comparison of various methods of analysis.	2008	Profile case	Evaluation of healthcare delivery	*N* = 60
Flynn TN, Louviere JJ, Marley AAJ, Coast J, Peters TJ [95]	Rescaling quality of life values from discrete choice experiments for use as QALYs: a cautionary tale	2008	Profile case	Evaluation of quality of life	*N* = 478
Swancutt DR, Greenfield, SM Wilson S [96]	Women’s colposcopy experience and preferences: a mixed methods study	2008	Profile case	Evaluation of diagnosis/screening interventions	N/A (Aim to achieve *N* = 100)

## References

[1] Johnson FR, Mohamed AF, Özdemir S, Marshall DA, Phillips KA (2011). How does cost matter in health‐care discrete‐choice experiments?. Health Econ.

[2] Johnson RF, Lancsar E, Marshall D, Kilambi V, Mühlbacher A, Regier DA (2013). Constructing experimental designs for discrete-choice experiments: report of the ISPOR conjoint analysis experimental design good research practices task force. Value Health.

[3] Mc Neil Vroomen J, Zweifel P (2011). Preferences for health insurance and health status: does it matter whether you are dutch or german?. Eur J Health Econ.

[4] Mühlbacher A, Bethge S, Tockhorn A (2013). Präferenzmessung im gesundheitswesen: grundlagen von discrete-choice-experimenten. Gesundheitsökonomie & Qualitätsmanagement.

[5] Telser H, Zweifel P (2007). Validity of discrete-choice experiments evidence for health risk reduction. Appl Econ.

[6] Mühlbacher A, Zweifel P, Kaczynski A, Johnson FR. Experimental Measurement of Preferences in Health Care Using Best-Worst Scaling (BWS): Theoretical and Statistical Issues. Health Economics Review; 2016. 10.1186/s13561-015-0077-z PMC4731383 26822869

[7] Thurstone LL (1994). A law of comparative judgment. Psychol Rev.

[8] Hensher DA, Rose JM, Greene WH. Applied choice analysis: a primer. Cambridge: Cambridge University Press; 2005.

[9] Marschak J . Binary Choice Constraints on Random Utility Indicators. No. 74. Cowles Foundation for Research in Economics, Yale University, 1959.

[10] Luce RD (1959). Individual choice behavior a theoretical analysi.

[11] McFadden D, Zarembka P (1974). Conditional logit analysis of qualitative choice behavior. Frontiers in econometrics.

[12] McFadden D (1986). The choice theory approach to market research. Mark Sci.

[13] Lancsar E, Louviere J. Estimating individual level discrete choice models and welfare measures using best-worst choice experiments and sequential best-worst MNL. Sydney: University of Technology, Centre for the Study of Choice (Censoc). 2008:1–24.

[14] Louviere JJ, Flynn TN (2010). Using best-worst scaling choice experiments to measure public perceptions and preferences for healthcare reform in Australia. Patient.

[15] Flynn TN (2010). Valuing citizen and patient preferences in health: recent developments in three types of best–worst scaling. Expert Rev Pharmacoecon Outcomes Res.

[16] Finn A, Louviere JJ (1992). Determining the appropriate response to evidence of public concern: the case of food safety. J Public Policy Mark.

[17] Marley AAJ, Flynn TN, Louviere JJ (2008). Probabilistic models of set-dependent and attribute-level best–worst choice. J Math Psychol.

[18] Marti J (2012). A best-worst scaling survey of adolescents’ level of concern for health and non-health consequences of smoking. Soc Sci Med.

[19] Helm R, Steiner M (2008). Präferenzmessung: Methodengestützte Entwicklung zielgruppenspezifischer Produktinnovationen.

[20] Telser H (2002). Nutzenmessung im Gesundheitswesen. Die Methode der Discrete-Choice-Experimente.

[21] Johnson RM, Orme BK (1996). How many questions should you ask in choice-based conjoint studies.

[22] Chrzan K, Orme B. An overview and comparison of design strategies for choice-based conjoint analysis. Sequium, WA: Sawtooth Software Research Paper Series. 2000.

[23] Kuhfeld WF. Marketing research methods in SAS. Experimental Design, Choice, Conjoint, and Graphical Techniques. Cary, NC: SAS-Institute TS-722. 2009.

[24] Smith NF, Street DJ (2003). The use of balanced incomplete block designs in designing randomized response surveys. Aust N Z J Statistics.

[25] Cochram WG, Cochran, Cox B. Experimental design. Hoboken, NJ: Wilex Classics Library; 1992.

[26] Louviere JJ, Hensher DA, Swait JD (2000). Stated choice methods: analysis and applications.

[27] Burgess L, Street DJ (2005). Optimal designs for choice experiments with asymmetric attributes. J Stat Planning and Inference.

[28] Coltman TR, Devinney TM, Keating BW (2011). Best–worst scaling approach to predict customer choice for 3PL services. J Bus Logist.

[29] Crouch GI, Louviere JJ. International Convention Site Selection: A further analysis of factor importance using best-worst scaling. Queensland: CRC for Sustainable Tourism; 2007.

[30] Flynn TN, Louviere JJ, Peters TJ, Coast J (2007). Best–worst scaling: what it can do for health care research and how to do it. J Health Econ.

[31] Flynn TN, Louviere JJ, Peters TJ, Coast J (2008). Estimating preferences for a dermatology consultation using best-worst scaling: Comparison of various methods of analysis. BMC Med Res Methodol.

[32] Wirth R (2010). Best-worst choice-based conjoint-analyse: Eine neue variante der wahlbasierten conjoint-analyse.

[33] Flynn T, Marley A. 8 Best-worst scaling: theory and methods. In: Hess S, Daly A, editors. Handbook of Choice Modelling. Cheltenham, UK: Edward Elgar Publishing; 2014. p. 178–201.

[34] Marley AAJ (2009). The best-worst method for the study of preferences: theory and application 2009: Working paper.

[35] Hoyos D (2010). The state of the art of environmental valuation with discrete choice experiments. Ecol Econ.

[36] McFadden D, Train K (2000). Mixed MNL models for discrete response. J Appl Econ.

[37] Rischatsch M, Zweifel P (2013). What do physicians dislike about managed care? Evidence from a choice experiment. Eur J Health Econ.

[38] Train KE. Discrete choice methods with simulation. Berkeley: Cambridge university press; 2009.

[39] Long JS, Freese J. Regression models for categorical dependent variables using Stata. Second edition. College Station, Texas: Stata press, 2006.

[40] Cohen S (2003). Maximum difference scaling: improved measures of importance and preference for segmentation.

[41] Vermunt JK, Magidson J. Latent class cluster analysis. In: Hagenaars JA, McCutchen AL, editors. Applied latent class analysis. Cambridge et al.: Cambrige University Press; 2002. p. 89–106.

[42] Orme B. Maxdiff analysis: Simple counting, individual-level logit, and hb. Sequim, WA: Sawtooth Software. 2009.

[43] Ratcliffe J, Couzner L, Flynn T, Sawyer M, Stevens K, Brazier J (2011). Valuing child health utility 9D health states with a young adolescent sample. Appl Health Econ Health Policy.

[44] Günther OH, Kürstein B, Riedel‐Heller SG, König HH (2010). The role of monetary and nonmonetary incentives on the choice of practice establishment: a stated preference study of young physicians in Germany. Health Serv Res.

[45] Chrzan K, Golovashkina N (2006). An empirical test of six stated importance measures. Int J Mark Res.

[46] Severin F, Schmidtke J, Mühlbacher A, Rogowski W (2013). Eliciting preferences for priority setting in genetic testing: a pilot study comparing best-worst scaling and discrete-choice experiments. Eur J Hum Genet.

[47] Bacon L, Lenk P, Seryakova K, Veccia E (2008). Comparing apples to oranges. Mark Res.

[48] Johnson FR, Yang J-C, Mohammed AF, editors. In Defense of Imperfect Experimental Designs: Statistical Efficiency and Measurement Error in Choice-Format Conjoint Analysis. Orlando, FL: Proceedings of the Sawtooth Software Conference 2012.

[49] Yang J.-C., Johnson FR, Kilambi V, Mohammed AF. Sample Size and Estimate Precision in Discrete-Choice Experiments: A Meta-Simulation Approach. Journal of Choice Modelling (in review). 2014.

[50] Beusterien K, Kennelly MJ, Bridges JF, Amos K, Williams MJ, Vasavada S (2015). Use of best-worst scaling to assess patient perceptions of treatments for refractory overactive bladder. Neurourol Urodyn.

[51] Flynn TN, Huynh E, Peters TJ, Al-Janabi H, Clemens S, Moody A (2015). Scoring the Icecap-a capability instrument. Estimation of a UK general population tariff. Health Econ.

[52] Franco MR, Howard K, Sherrington C, Ferreira PH, Rose J, Gomes JL (2015). Eliciting older people’s preferences for exercise programs: a best-worst scaling choice experiment. J Physiother.

[53] Gallego G, Dew A, Lincoln M, Bundy A, Chedid RJ, Bulkeley K (2015). Should I stay or should I go? Exploring the job preferences of allied health professionals working with people with disability in rural Australia. Hum Resour Health.

[54] Hashim H, Beusterien K, Bridges JP, Amos K, Cardozo L (2015). Patient preferences for treating refractory overactive bladder in the UK. Int Urol Nephrol.

[55] Hollin IL, Peay HL, Bridges JF (2015). Caregiver preferences for emerging duchenne muscular dystrophy treatments: a comparison of best-worst scaling and conjoint analysis. Patient.

[56] Meyfroidt S, Hulscher M, De Cock D, Van der Elst K, Joly J, Westhovens R (2015). A maximum difference scaling survey of barriers to intensive combination treatment strategies with glucocorticoids in early rheumatoid arthritis. Clin Rheumatol.

[57] Morrison W, Womer J, Nathanson P, Kersun L, Hester DM, Walsh C (2015). Pediatricians’ experience with clinical ethics consultation: a national survey. J Pediatr.

[58] Muhlbacher AC, Bethge S, Kaczynski A, Juhnke C (2015). Objective criteria in the medicinal therapy for type II diabetes: An analysis of the patients’ perspective with analytic hierarchy process and best-worst scaling. Gesundheitswesen.

[59] Narurkar V, Shamban A, Sissins P, Stonehouse A, Gallagher C (2015). Facial treatment preferences in aesthetically aware women. Dermatol Surg.

[60] O’Hara NN, Roy L, O’Hara LM, Spiegel JM, Lynd LD, FitzGerald JM (2015). Healthcare worker preferences for active tuberculosis case finding programs in South Africa: A best-worst scaling choice experiment. PLoS One.

[61] Peay H, Hollin IL, Bridges JFP (2015). Prioritizing parental worry associated with Duchenne Muscular Dystrophy using Best-Worst Scaling. J Genet Counsel.

[62] Ratcliffe J, Huynh E, Stevens K, Brazier J, Sawyer M, Flynn T (2015). Nothing about us without us? A comparison of adolescent and adult health-state values for the child health utility-9D using profile case Best-Worst Scaling. Health Econ.

[63] Ross M, Bridges JF, Ng X, Wagner LD, Frosch E, Reeves G (2015). A best-worst scaling experiment to prioritize caregiver concerns about ADHD medication for children. Psychiatr Serv.

[64] Wittenberg E, Bharel M, Saada A, Santiago E, Bridges JF, Weinreb L (2015). Measuring the preferences of homeless women for cervical cancer screening interventions: development of a best-worst scaling survey. Patient.

[65] Yan K, Bridges JF, Augustin S, Laine L, Garcia-Tsao G, Fraenkel L (2015). Factors impacting physicians’ decisions to prevent variceal hemorrhage. BMC Gastroenterol.

[66] Damery S, Biswas M, Billingham L, Barton P, Al-Janabi H, Grimer R (2014). Patient preferences for clinical follow-up after primary treatment for soft tissue sarcoma: a cross-sectional survey and discrete choice experiment. Eur J Surg Oncol.

[67] dosReis S, Ng X, Frosch E, Reeves G, Cunningham C, Bridges JF (2014). Using best-worst scaling to measure caregiver preferences for managing their child’s adhd: a pilot study. Patient.

[68] Ejaz A, Spolverato G, Bridges JF, Amini N, Kim Y, Pawlik TM (2014). Choosing a cancer surgeon: analyzing factors in patient decision making using a best-worst scaling methodology. Ann Surg Oncol.

[69] Hauber AB, Mohamed AF, Johnson FR, Cook M, Arrighi HM, Zhang J (2014). Understanding the relative importance of preserving functional abilities in Alzheimer’s disease in the United States and Germany. Qual Life Res.

[70] Hofstede SN, van Bodegom-Vos L, Wentink MM, Vleggeert-Lankamp CL, Vliet Vlieland TP, de Mheen PJ M-v (2014). Most important factors for the implementation of shared decision making in sciatica care: ranking among professionals and patients. PLoS One.

[71] Peay HL, Hollin I, Fischer R, Bridges JF (2014). A community-engaged approach to quantifying caregiver preferences for the benefits and risks of emerging therapies for Duchenne muscular dystrophy. Clin Ther.

[72] Roy L, Bansback N, Marra C, Carr R, Chilvers M, Lynd L (2014). Evaluating preferences for long term wheeze following RSV infection using TTO and best-worst scaling. All Asth Clin Immun.

[73] Torbica A, De Allegri M, Belemsaga D, Medina-Lara A, Ridde V (2014). What criteria guide national entrepreneurs’ policy decisions on user fee removal for maternal health care services? Use of a best-worst scaling choice experiment in West Africa. J Health Serv Res Policy.

[74] Ungar WJ, Hadioonzadeh A, Najafzadeh M, Tsao NW, Dell S, Lynd LD (2014). Quantifying preferences for asthma control in parents and adolescents using best-worst scaling. Respir Med.

[75] van Til J, Groothuis-Oudshoorn C, Lieferink M, Dolan J, Goetghebeur M (2014). Does technique matter; a pilot study exploring weighting techniques for a multi-criteria decision support framework. Cost Eff Resour Alloc.

[76] Whitty JA, Ratcliffe J, Chen G, Scuffham PA (2014). Australian public preferences for the funding of new health technologies: a comparison of discrete choice and profile case best-worst scaling methods. Med Decis Making.

[77] Whitty JA, Walker R, Golenko X, Ratcliffe J (2014). A think aloud study comparing the validity and acceptability of discrete choice and best worst scaling methods. PLoS One.

[78] Xie F, Pullenayegum E, Gaebel K, Oppe M, Krabbe PF (2014). Eliciting preferences to the EQ-5D-5 L health states: discrete choice experiment or multiprofile case of best-worst scaling?. Eur J Health Econ.

[79] Xu F, Chen G, Stevens K, Zhou H, Qi S, Wang Z (2014). Measuring and valuing health-related quality of life among children and adolescents in mainland China--a pilot study. PLoS One.

[80] Yuan Z, Levitan B, Burton P, Poulos C, Brett Hauber A, Berlin JA (2014). Relative importance of benefits and risks associated with antithrombotic therapies for acute coronary syndrome: patient and physician perspectives. Curr Med Res Opin.

[81] Yoo HI, Doiron D (2013). The use of alternative preference elicitation methods in complex discrete choice experiments. J Health Econ.

[82] Gallego G, Bridges JF, Flynn T, Blauvelt BM, Niessen LW (2012). Using best-worst scaling in horizon scanning for hepatocellular carcinoma technologies. Int J Technol Assess Health Care.

[83] Knox S, Viney R, Street D, Haas M, Fiebig D, Weisberg E (2012). What’s good and bad about contraceptive products?. PharmacoEconomics.

[84] Molassiotis A, Emsley R, Ashcroft D, Caress A, Ellis J, Wagland R (2012). Applying best-worst scaling methodology to establish delivery preferences of a symptom supportive care intervention in patients with lung cancer. Lung Cancer.

[85] Netten A, Burge P, Malley J, Potoglou D, Towers AM, Brazier J (2012). Outcomes of social care for adults: developing a preference-weighted measure. Health Technol Assess.

[86] Ratcliffe J, Flynn T, Terlich F, Stevens K, Brazier J, Sawyer M (2012). Developing adolescent-specific health state values for economic evaluation: an application of profile case best-worst scaling to the child health utility 9D. PharmacoEconomics.

[87] van der Wulp I, van den Hout WB, de Vries M, Stiggelbout AM, van den Akker-van Marle EM (2012). Societal preferences for standard health insurance coverage in the Netherlands: a cross-sectional study. BMJ Open.

[88] Al-Janabi H, Flynn TN, Coast J (2011). Estimation of a preference-based carer experience scale. Med Decis Making.

[89] Kurkjian TJ, Kenkel JM, Sykes JM, Duffy SC (2011). Impact of the current economy on facial aesthetic surgery. Aesthet Surg J.

[90] Rudd M (2011). An exploratory analysis of societal preferences for research-driven quality of life improvements in Canada. Soc Indic Res.

[91] Simon A (2011). Patient involvement and information preferences on hospital quality: results of an empirical analysis. Unfallchirurg.

[92] van Hulst LT, Kievit W, van Bommel R, van Riel PL, Fraenkel L (2011). Rheumatoid arthritis patients and rheumatologists approach the decision to escalate care differently: results of a maximum difference scaling experiment. Arthritis Care Research.

[93] Wang T, Wong B, Huang A, Khatri P, Ng C, Forgie M (2011). Factors affecting residency rank-listing: a Maxdiff survey of graduating Canadian medical students. BMC Med Educ.

[94] Imaeda A, Bender D, Fraenkel L (2010). What is most important to patients when deciding about colorectal screening?. J Gen Intern Med.

[95] Flynn T, Louviere J, Marley A, Coast J, Peters T (2008). Rescaling quality of life values from discrete choice experiments for use as QALYs: a cautionary tale. Popul Health Metrics.

[96] Swancutt D, Greenfield S, Wilson S (2008). Women’s colposcopy experience and preferences: a mixed methods study. BMC Womens Health.

